# Inhibition of cystathionine β-synthase promotes apoptosis and reduces cell proliferation in chronic myeloid leukemia

**DOI:** 10.1038/s41392-020-00410-5

**Published:** 2021-02-08

**Authors:** Dan Wang, Huan Yang, Yun Zhang, Rong Hu, Dongjie Hu, Qunxian Wang, Yannan Liu, Mingjing Liu, Zijun Meng, Weihui Zhou, Weihong Song

**Affiliations:** 1 Chongqing City Key Lab of Translational Medical Research in Cognitive Development and Learning and Memory Disorders; Ministry of Education Key Laboratory of Child Development and Disorders; National Clinical Research Center for Child Health and Disorders (Chongqing); and Department of Neurology, Children’s Hospital of Chongqing Medical University, 400014 Chongqing, China; 2 Townsend Family Laboratories, Department of Psychiatry, The University of British Columbia, 2255 Wesbrook Mall, Vancouver, BC V6T 1Z3 Canada

**Keywords:** Haematological cancer, Haematological cancer

## Abstract

Increased endogenous hydrogen sulfide (H_2_S) level by cystathionine β-synthase (CBS) has been shown to closely relate tumorigenesis. H_2_S promotes angiogenesis, stimulates bioenergy metabolism and inhibits selective phosphatases. However, the role of CBS and H_2_S in chronic myeloid leukemia (CML) remains elusive. In this study, we found that CBS and H_2_S levels were increased in the bone marrow mononuclear cells of pediatric CML patients, as well as in the CML-derived K562 cells and CBS expression levels were correlated with different disease phases. Inhibition of CBS reduced the proliferation of the CML primary bone marrow mononuclear cells and induced growth inhibition, apoptosis, cell cycle arrest, and migration suppression in K562 cells and tumor xenografts. The knockdown of CBS expression by shRNA and inhibiting CBS activity by AOAA decreased the endogenous H_2_S levels, promoted mitochondrial-related apoptosis and inhibited the NF-κB-mediated gene expression. Our study suggests that inhibition of CBS induces cell apoptosis, as well as limits cell proliferation and migration, a potential target for the treatment of chronic myeloid leukemia.

## Introduction

Chronic myeloid leukemia (CML) is a myeloproliferative malignant clonal disease with abnormal chromosome formation^1,2^ that causes the formation of the breakpoint cluster region *bcr-abl.*^3^ The resulting BCR-ABL fusion protein has abnormal tyrosine kinase activity, leading to the abnormal activation of the downstream molecules and the formation of the malignant clones.^4,5^ Although the tyrosine kinase inhibitors greatly improve CML prognosis,^6–8^ there are many issues limiting their broader clinical applications, including the fusion protein formation, relapse after drug withdrawal, toxic side effects and the primary and secondary resistance.^9^ Therefore, alternative therapeutic strategies such as new anti-proliferative and/or pro-apoptotic compounds are proposed for treating CML.^10^

Hydrogen sulfide (H_2_S), produced in living organisms, plays an important pathophysiological role in the cardiovascular system, central nervous system, gastrointestinal tract and other tissues and organs.^11–14^ Recently, the relationship between H_2_S and tumors has attracted great attention.^15,16^ Growing evidence shows that CBS-induced endogenous H_2_S production plays an important role in promoting energy synthesis, proliferation and invasion of tumor cells in different kinds of cancers.^15–17^ Szabo et al. reported that the expressions of CBS and H_2_S were significantly higher in colorectal cancer tissues than those in the normal mucosal area adjacent to the cancer tissue.^18^ Inhibition of CBS activity or CBS expression reduced H_2_S levels, which was associated with the inhibition of cell proliferation and a significant reduction in the growth rate of tumor tissue xenografts. Similar findings have also been found in other cancers including ovarian cancer, prostate cancer, and liver cancer.^19–22^ The potential mechanisms underlying the effects include the promotion of energy metabolism,^23^ angiogenesis,^24^ and the regulation of specific kinase proteins such as PTEN and PTP1B.^25,26^

CML in children is relatively rare. In this study, we found that CBS expression and endogenous H_2_S production were increased in the pediatric CML patients. Furthermore, reduction of H_2_S production by inhibition of CBS expression or activity stimulated leukemia cell apoptosis and inhibited cell proliferation by downregulating NF-κB activity. Our work demonstrates that inhibition of CBS has therapeutic effect on CML.

## Results

### Increased CBS expression in CML

**Fig. 1:**
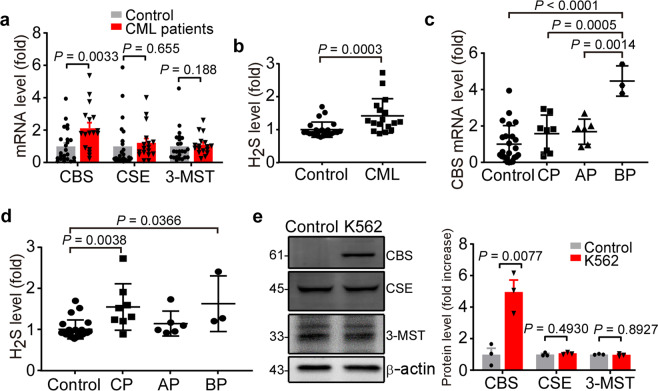
CBS and H2S levels were increased in pediatric CML patients and CML-derived K562 cells. The CBS, CSE, and 3-MST mRNA levels (**a**) and bone marrow H_2_S levels (**b**) in CML patients (*n* = 17) and the controls (*n* = 24) were measured by RT-PCR and methylene blue assay, respectively. The CBS mRNA levels (**c**) and bone marrow H_2_S levels (**d**) in the controls (*n* = 24) and different stages of CML (*n* = 8 of chronic phase, *n* = 6 of accelerated phase, *n* = 3 of blastic phase). **e** CD34^+^ umbilical cord hematopoietic stem cells were extracted from the neonatal cord blood. The protein levels of CBS, CSE, and 3-MST in CD34+ cells and CML-derived K562 cells were detected by Western blot (*n* = 3). The results are expressed as mean ± SEM. Statistical significance was determined by Mann–Whitney test, ANOVA followed by Tukey’s multiple comparisons test or two-tailed Student’s *t* test

In mammals, hydrogen sulfide (H_2_S) is mainly produced by three enzymes including cystathionine β-synthase (CBS), cystathionine γ-synthase (CSE) and 3-mercaptopyruvate sulfurtransferase (3-MST).^27^ To examine the expressions of these three enzymes and H_2_S in bone marrow of the newly diagnosed CML pediatric patients (Table 1) and CML-derived cell line K562, RT-PCR, Western blot, and methylene blue method were performed. The results showed that the CBS mRNA level was significantly increased in CML patients by 2.13 ± 0.34 fold compared with the control groups (*P* = 0.0033), while the levels of CSE and 3-MST had no significant differences between the two groups (Fig. 1a). The H_2_S level was also markedly elevated in the bone marrow of CML patients by 1.42 ± 0.13 fold (*P* = 0.0003) (Fig. 1b). The increased level of the CBS expression in the chronic phase (CP) (1.58 ± 0.36, *P* = 0.0005) and the accelerated phase (AP) (1.69 ± 0.28, *P* = 0.0014) were much lower than that in the blastic phase (BP) (4.47 ± 0.48) (Fig. 1c). However, the level of H_2_S was not related to the disease stage (CP = 1.55 ± 0.20, AP = 1.14 ± 0.12, BP = 1.63 ± 0.39, *P* > 0.05) (Fig. 1d). Similarly, CML-derived K562 cells showed significantly higher CBS expression than that in human CD34+ umbilical cord hematopoietic stem cells as the control (1.00 ± 0.39 vs. 4.97 ± 0.74, *P* = 0.0077), while the expressions of CSE and 3-MST did not have significant changes (Fig. 1e). These results suggest that CBS and H_2_S may play a role in CML.

**Table 1:** Clinical characteristics of the patients

	CP (*n* = 8)	AP (*n* = 6)	BP (*n* = 3)	Total
Age (years), median (range)	8.4 (6.7–10.9)	12.2 (9.8–14.8)	8.9 (1.8–11.8)	9.8 (7.0–11.8)
Blast percentage/median (range)	1.0 (1.0–2.5)	11.5 (4.5–17.5)	42.5 (35.0–67.5)	–
Male/female (*n*/*n*)	4/4	3/3	1/2	8/9
White blood cells × 10^9^/median (range)	316.46 (134.51–403.66)	233.58 (212.94–429.89)	39.03 (2.10–563.69)	240.77 (113.02–427.20)
Platelet count × 10^9^/median (range)	308.0 (258.0–09.5)	293.0 (115.0–329.0)	47.0 (45.0–256.0)	285.0 (115.0–329.0)
Hemoglobin level (g/L)/median (range)	81 (79–90)	79 (77–92)	85 (66–91)	81 (78–91)

### Inhibition of CBS activity induces apoptosis and reduces cell growth

**Fig. 2:**
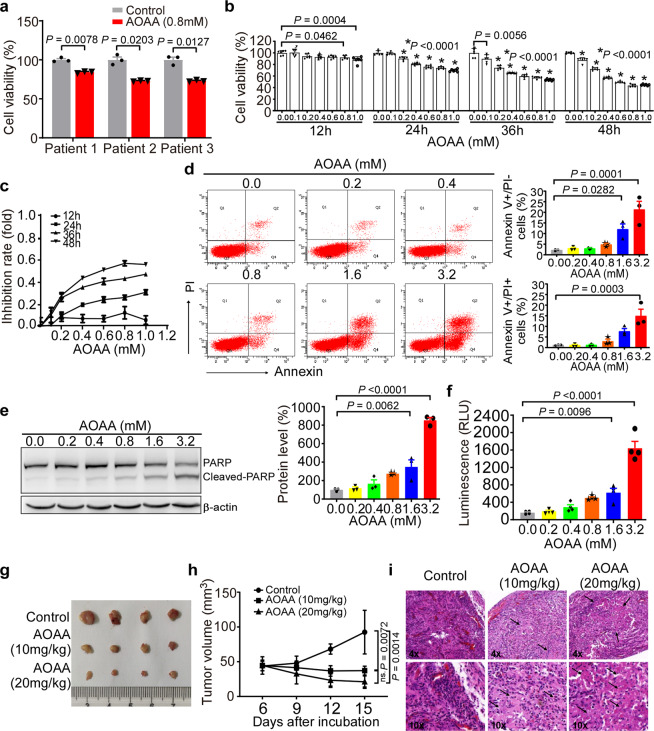
AOAA treatment induced cell apoptosis and inhibited cell growth. **a** Peripheral blood mononuclear cells were extracted from three CML patients and treated with AOAA (0.8 mM) for 48 h. Cell viability was detected by MTT assay (*n* = 3). **b** K562 cells were treated with AOAA at different concentrations (0.0, 0.1, 0.2, 0.4, 0.6, 0.8, and 1.0 mM for 12, 24, 36, and 48 h). Cell viability was measured by MTT (*n* = 6). **c** The inhibition rates are shown in the line chart. **d** K562 cells were treated with AOAA at 0.0, 0.2, 0.4, 0.8, 1.6, and 3.2 mM for 48 h. The cells were stained with Annexin V-FITC/PI and analyzed by flow cytometry. The percentage of Annexin V+/PI- cells, which represent early apoptotic cells, was calculated (*n* = 3). **e** K562 cells treated with AOAA at different concentrations (0.0, 0.2, 0.4, 0.8, 1.6, 3.2 mM) were analyzed by Western blotting to evaluate the expression levels of PARP and cleaved-PARP (*n* = 3). **f** Caspase 3/7 activity was measured by chemiluminescence (*n* = 4). **g** The images of tumor xenografts isolated from the mice treated with vehicle solution and AOAA (10 or 20 mg/kg). **h** Quantification of tumor volume. **i** H & E staining of the tumor grafts. Black arrows indicate necrotic lesions. The results are expressed as mean ± SEM. Two-tailed Student’s *t* test was used to analyze the difference between 2 groups, and multiple comparisons were analyzed by ANOVA followed by Tukey’s multiple comparisons test

To examine the effect of CBS on cell viability, the MTT assay was performed. Bone marrow mononuclear cells from the CML patients were treated with 0.8 mM aminooxyacetic acid (AOAA) for 48 h, which has been shown to inhibit CBS activity.^28^ AOAA significantly reduced the cell viability to 84.43 ± 0.70% (*P* = 0.0078), 73.18 ± 0.40% (*P* = 0.0203), and 73.21 ± 0.66% (*P* = 0.0127), respectively, in each patient compared with the controls (*P* < 0.05) (Fig. 2a). Next we treated CML-derived leukemia cell line K562 with the CBS inhibitor. The results showed that AOAA decreased the cell viability in a concentration-dependent and time-dependent manner compared to that in the controls (*P* < 0.05) (Fig. 2b, c). In contrast, the percentage of annexin V+/PI− apoptotic cells was significantly increased after treatment with AOAA for 48 h compared to the controls (12.1 ± 2.43% at 1.6 mM, *P* = 0.0282 and 21.37 ± 3.85% at 3.2 mM vs. 2.23 ± 0.22% at 0 mM, *P* = 0.0001). The percentage of annexin V+/PI+ apoptotic cells increased to 14.93 ± 3.14% after treatment with 3.2 mM AOAA for 48 h (Fig. 2d). Furthermore, treatment with 1.6 or 3.2 mM of AOAA increased the level of the cleaved-PARP to 348.4 ± 76.16% (*P* = 0.0062) and 852.6 ± 30.27% (*P* < 0.0001), respectively (Fig. 2e) and enhanced caspase 3/7 activity to 624.8 ± 95.45RLU (*P* = 0.0096) and 1646 ± 152.8RLU (*P* < 0.0001), respectively (Fig. 2f). To examine the effect of AOAA on tumor cell growth in vivo, we generated a tumor mouse model by subcutaneously injecting K562 cells in athymic BALB/nu mice. The mice with palpable tumors were treated with 10 or 20 mg/kg of AOAA daily for 9 days, while control mice received vehicle solution. AOAA treatment significantly inhibited tumor growth in K562-xenograft tumor mice relative to the vehicle-injected group (Fig. 2g). Quantification showed that treatment with 10 or 20 mg/kg reduced tumor volumn to 40.18 ± 3.46% (*P* = 0.0072) and 22.84 ± 4.55% (*P* = 0.0014), respectively, (Fig. 2h). In addition, tumor tissues of the control mice showed the pathological hallmarks of stable-phase CML with immature granulocyte and megakaryocyte precursors and angiogenesis. In contrast, AOAA treatment destroyed the structure of tumor cells and induced cell death (Fig. 2i).

**Fig. 3:**
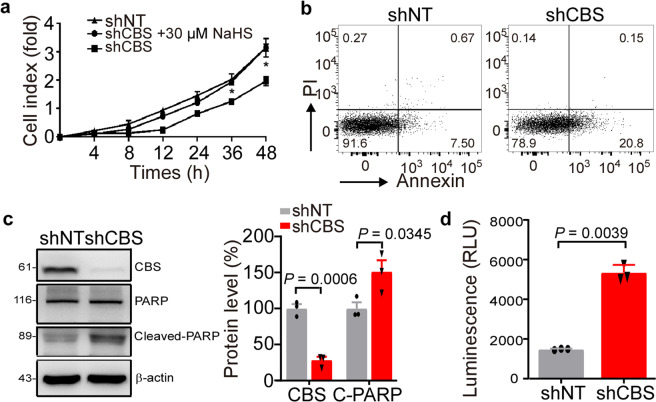
Knockdown of CBS expression by shRNA increased K562 cell apoptosis. **a** K562 cells were transfected with CBS-shRNA lentiviral vector (shCBS) or a non-targeting sequence vector as the control (shNT). The transfected cells were then treated with or without NaHS (30 μM). The cell viability was detected by performing the MTT assay (*n* = 3). **b** shNT and shCBS transfected K562 cells were stained with Annexin V-APC/PI and subjected to flow cytometry analysis (*n* = 3). **c** Western blot analysis was performed to detect the expression of CBS and PARP in the shNT and shCBS transfected K562 cells (*n* = 3). **d** Caspase 3/7 activity was measured by chemiluminescence (*n* = 3). The results are expressed as mean ± SEM. Two-tailed Student’s *t* test was used to analyze the difference between 2 groups, and multiple comparisons were analyzed by ANOVA followed by Tukey’s multiple comparisons test. *P < 0.05

To further determine whether the effect of AOAA on K562 cells is CBS-dependent, a lentivirus vector carrying a *CBS* gene specific short hairpin RNA (shRNA) sequence was used to silence CBS expression in K562 cells. A non-targeted sequence in lentivirus (shNT) was used as a control. Compared with the shNT-infected group, the proliferation rate was reduced in shCBS-infected K562 cells, and the inhibitory effect was rescued when exogenous H_2_S was applied (*P* < 0.05) (Fig. 3a). The number of annexin V+/PI− apoptotic cells was also increased in the shCBS-infected K562 cells compared to the controls (14.00 ± 0.97 vs. 7.96 ± 0.78, *P* < 0.05) (Fig. 3b). The expression of CBS in K562 cells was decreased by 70% in the shCBS-infected K562 cells compared to the control (*P* < 0.05) (Fig. 3c). The levels of cleaved-PARP (Fig. 3c) and caspase 3/7 activity (Fig. 3d) were significantly increased compared to those in the control (100 ± 8.371 compared to 150.90 ± 15.67 and 1482 ± 20.51 compared to 5350 ± 222.5, respectively) (*P* < 0.05). These results suggest that inhibition of CBS decreases proliferation and induces apoptosis both in the bone marrow mononuclear cells of the CML patients and the CML-derived K562 cells.

### Knockdown of CBS expression in K562 induces S-phase arrest and reduces cell migration

**Fig. 4:**
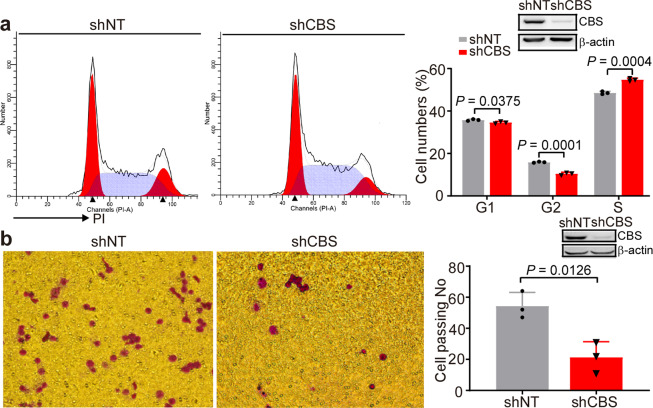
Knockdown of CBS expression in K562 cells induced S-phase arrest and inhibited cell migration. **a** After knocking down the CBS expression, the cell cycle was analyzed with the PI staining and the number of cells in each phase was analyzed by Motfit (*n* = 3). **b** Transwell assays were performed to evaluate the migration abilities of shNT-K562 and shCBS-K562 cells (*n* = 3). The results are expressed as mean ± SEM. Statistical significance was determined by two-tailed Student’s *t* test

To further study the biological role of CBS in CML, flow cytometry was performed to examine the cell cycle of K562 cells (Fig. 4a). We found that CBS-shRNA significantly reduced the number of K562 cells in G2/M phase (15.89 ± 0.16% compared to 10.5 ± 0.35%, *P* = 0.0001) and increased the number of cells in S phase (48.58 ± 00.39% compared to 54.83 ± 0.41%, *P* = 0.0004), indicating that the cell cycle was arrested in the S phase. Next, we examined cell migration by counting number of the cells transmigrated through the transwell after shRNA treatment. Cell migration assay showed that the migration of K562 cells in the CBS-shRNA group was significantly lower than that in the shNT control group (21.33 ± 5.78 compared to 54.33 ± 5.04, *P* = 0.0126) (Fig. 4b).

### Inhibition of CBS reduces H2S production

**Fig. 5:**
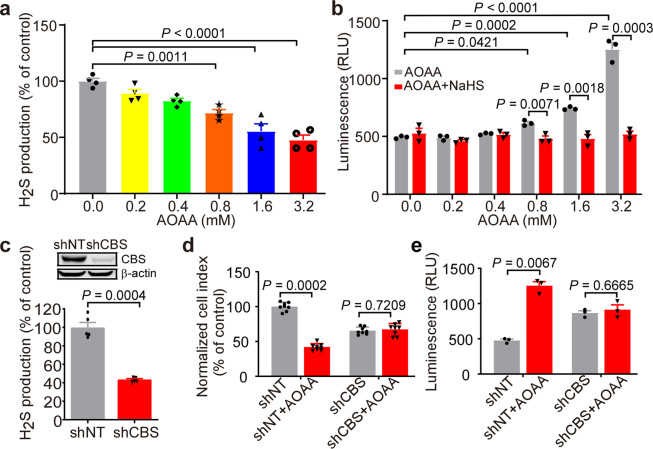
Inhibition of CBS reduced endogenous H2S generation. **a** K562 cells were treated with AOAA (0.0, 0.2, 0.4, 0.8, 1.6, and 3.2 mM) for 48 h. Endogenous levels of H_2_S were detected by fluorescence assays (ex = 365 nm, em = 450 nm). **b** K562 cells were treated with different concentrations of AOAA for 48 h with or without NaHS (30 μM), caspase 3/7 activity was tested by using chemiluminescence method (*n* = 3). **c** The K562 cells were transfected with CBS-shRNA lentiviral vector (shCBS) or the control group (shNT) and the level of H_2_S was analyzed (*n* = 5). shNT and shCBS cells were treated with or not 1 mM AOAA for 48 h, cell viability (*n* = 6) and caspase 3/7 activity (*n* = 3) were detected by MTT (**d**) or chemiluminescence method (**e**), respectively. The results are expressed as mean ± SEM. Two-tailed Student’s *t* test was used to analyze the difference between 2 groups, and multiple comparisons were analyzed by ANOVA followed by Tukey’s multiple comparisons test

Previous studies have found that CBS and H_2_S play important roles in promoting tumor cell proliferation.^18,20,29,30^ To determine that the anti-proliferation and pro-apoptotic effect of CBS is mediated by H_2_S, K562 cells were treated with AOAA for 48 h, or transfected with the shRNA to knockdown CBS expression. H_2_S levels in the cells were analyzed.^31,32^ AOAA treatment significantly reduced the intracellular H_2_S levels in a concentration-dependent manner (71.8 ± 2.30, *P* = 0.0011; 55.39 ± 6.66, *P* < 0.0001; and 47.74 ± 4.29, *P* < 0.0001 after treatment of AOAA at 0.8, 1.6, and 3.2 mM, respectively) (Fig. 5a). In addition, treatment with AOAA markedly increased the activity of caspase3/7 that executes apoptosis in K562 cells (493.3 ± 6.69 to 615.7 ± 16.69, *P* = 0.0421; 741.7 ± 8.21, *P* = 0.0002; 1254 ± 57.04, *P* < 0.0001 after treatment of AOAA at 0.8, 1.6, and 3.2 mM, respectively). However, addition of 30 μm NaHS significantly inhibited AOAA’s effect (Fig. 5b). Endogenous H_2_S was also significantly reduced by knockdown of CBS in K562 cells with shRNA (43.75 ± 0.83 compared to 100 ± 5.31, *P* = 0.0004) (Fig. 5c). In addition, AOAA lost its anti-proliferative and pro-apoptotic effects in K562 cells with CBS silencing (Fig. 5d & e). Our data demonstrate that CBS maintains endogenous H_2_S level in CML-derived K562 cells, which plays an important role in the inhibition of cell proliferation and induction of cell apoptosis.

### Inhibition of CBS promotes apoptosis through the mitochondrial pathway

**Fig. 6:**
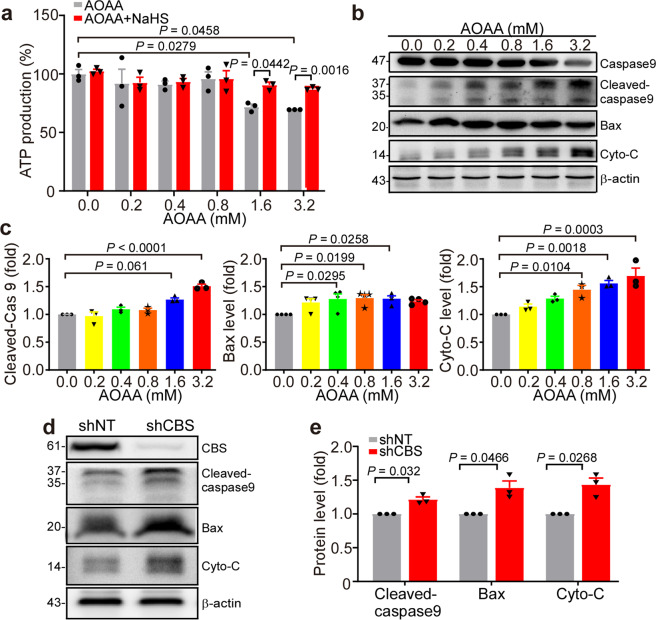
Inhibition of CBS induced apoptosis via the mitochondrial pathway. **a** K562 cells were treated with AOAA (0.0, 0.2, 0.4, 0.8, 1.6, and 3.2 mM) for 48 h with or without NaHS (30 μM). ATP production was measured by using chemiluminescence method (*n* = 3). The values of all groups are normalized with the non-intervention group (AOAA 0.0 mM). **b**, **c** K562 cells were treated with AOAA (0.0, 0.2, 0.4, 0.8, 1.6, and 3.2 mM) for 48 h. Western blot analysis was performed to detect the expressions of caspase 9/cleaved caspase-9, Bax and the cytosolic Cyto C proteins. **d**, **e** The expressions of cleaved caspase-9, Bax and cytoplasmic Cyto C proteins in shCBS- and shNT-K562 cells were detected by Western blot analysis (*n* = 3). The results are expressed as mean ± SEM. Two-tailed Student’s *t* test was used to analyze the difference between 2 groups, and multiple comparisons were analyzed by ANOVA followed by Tukey’s multiple comparisons test

CBS is mainly a cytosolic enzyme, but has been detected in mitochondria in specific cell types such as HCT116 cells to regulate mitochondrial function, cell proliferation and migration.^18^ Since CBS and H_2_S affect mitochondrial energy metabolism,^33^ we further measured the ATP production in the AOAA-treated K562 cells. The results showed that AOAA reduced ATP production in K562 cells, while treatment with NaHS restored the ATP levels (Fig. 6a). Next, we investigated the effect of CBS on mitochondrial apoptosis pathway. Compared to the controls, the levels of the cleaved-caspase 9 and Bax were significantly increased in the cells treated with different concentrations of AOAA for 48 h. The release of cytochrome C (Cyto C) into the cytoplasm was also significantly increased to 1.45 ± 0.08, *P* = 0.0104; 1.56 ± 0.05, *P* = 0.0018; 1.69 ± 0.14, *P* = 0.0003 with the treatment of 0.8, 1.6, and 3.2 mM AOAA (Fig. 6b, c). In addition, inhibition of CBS expression by shRNA also significantly increased the levels of cleaved-caspase 9, Bax and cytoplasmic Cyto C to 1.21 ± 0.04, *P* = 0.0320, 1.33 ± 0.073, *P* = 0.0466 and 1.46 ± 0.08, *P* = 0.0268, respectively (Fig. 6d, e). These results indicate that the CBS mediates cell apoptosis through mitochondrial pathway.

### Inhibition of CBS activity significantly reduces NF-κB signaling

BCR-ABL1 dimerization induces a self-phosphorylation to create a docking site, which interacts with an intermediate adapter protein such as GRB2 to activate multiple pathways including PI3K/AKT, MAPK, and ERK. These signaling pathways promote the cell survival and cell adhesion of hematopoietic stem cells, leading to form CML abnormal clones.^5,34,35^ To determine whether inhibition of CBS affects BCR-ABL expression or its phosphorylation, and the downstream PI3K/AKT, MAPK, ERK signaling pathways, Western blot analysis was performed to detect the phosphorylation levels of related proteins in the cells treated with different concentrations of AOAA or transfected with shRNA. The results showed that there was no significant difference of total AKT, phosphorylated AKT, phosphorylated P38, and phosphorylated ERK protein levels in the cells treated with different concentrations of AOAA (Supplementary Fig. S1a). Compared with the shNT control group, the knockdown of the CBS expression by shRNA had no significant effect on the levels of BCR-ABL, phosphorylated BCR-ABL, phosphorylated AKT, phosphorylated P38 and phosphorylated ERK (Supplementary Fig. S1b).

**Fig. 7:**
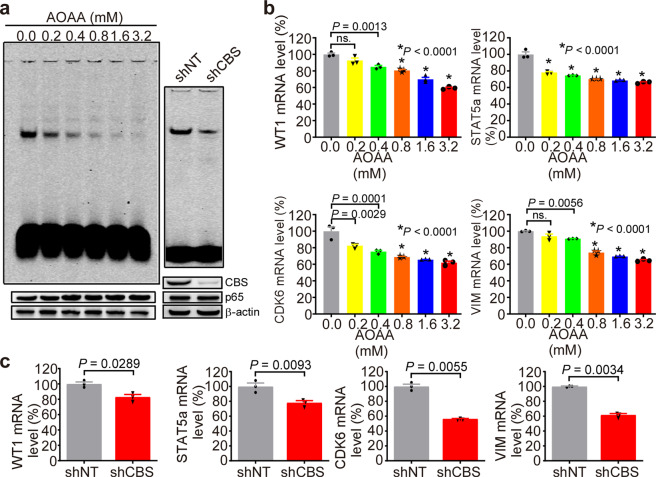
Inhibition of CBS decreased the transcriptional activity of NF-κB. K562 cells were treated with AOAA (0.0, 0.2, 0.4, 0.8, 1.6, and 3.2 mM) for 48 h or transfected with CBS-shRNA lentiviral vector (shCBS). **a** The binding of NF-κB to DNA was assayed by EMSA. **b**, **c** The mRNA levels of WT1, STAT5a, CDK6, and VIM were measured by RT-PCR (*n* = 3). The results are expressed as mean ± SEM. Two-tailed Student’s *t* test was used to analyze the difference between 2 groups, and multiple comparisons were analyzed by ANOVA followed by Tukey’s multiple comparisons test

The cysteine^36^ of NF-κB could bind to RPS3 by forming a disulfide bond, and the decrease of H_2_S affects the activity of NF-κB by reducing disulfide bond.^37^ To further investigate the effect of CBS on NF-κB activity, we examined NF-κB transcriptional activity by an electrophoretic mobility shift assay (EMSA) and RT-PCR. The activity of NF-κB was further confirmed by cold-probe competition experiments and supershift experiments (Supplementary Fig. S2). The results showed that the DNA-binding activity of NF-κB was significantly decreased with the increased concentration of AOAA or the knockdown of CBS expression (*P* < 0.05) (Fig. 7a). We further detected the mRNA levels of NF-κB-regulated genes including WT1, STAT5a, CDK6, and VIM, which play important roles in the development of leukemia.^36,38–41^ AOAA treatment (Fig. 7b) or shRNA transfection (Fig. 7c) significantly reduced the mRNA levels of these genes (*P* < 0.05). These results suggested that inhibition of CBS markedly decreased NF-κB signaling and its mediated target gene expression in K562 cells.

## Discussion

CML is a malignant clonal disease originating from hematopoietic stem cells. It has an incidence of 1–2/100,000 and accounts for approximately 15% of newly diagnosed leukemia.^42^ The chronic phase of the disease is characterized by atypical symptoms, such as weight loss, anemia, night sweats and splenomegaly. Most patients are diagnosed due to a high white blood cell count by a routine examination. If the patients do not accept an appropriate treatment, they will enter a period of higher malignancy with a relatively poor prognosis.^35^

To date, allogeneic hematopoietic stem cell transplantation (allo-HSCT) is widely accepted as the only effective approach to cure the disease.^43,44^ However, with the limitation of the donor availability and high medical expenses, only a small number of patients have received allo-HSCT. Although the tyrosine kinase inhibitors have greatly improved the overall prognosis of CML,^8^ there are many concerns, such as the drug tolerance and resistance.^45^ Accordingly, exploring novel approaches to treat this disease is required.

More than 95% of CML patients have a specific genetic abnormality in chromosome 22, the Philadelphia translocation (Ph) t(9;22)(q34;q11), which produces the breakpoint assembly region gene *bcr-abl*,^2,3^ The genetic defect creates a p210-Bcr-Abl fusion protein that activates the downstream pathways through its abnormal tyrosine kinase activity, resulting in the CML phenotypes.^4,5,46^ As a co-activated downstream molecule of these pathways, NF-κB has been extensively studied in BCR-ABL-related hematological tumors.^47–51^ NF-κB activity was significantly increased in human primary Ph+ leukemia during the stage of BP in CML;^52^ In addition, studies on other groups of hematological tumors (e.g., AML, CP of CML, and BP of CML) also found that NF-κB activity was significantly elevated during the stage of BP compared with the stage of CP.^53^ However, subsequent studies have shown that NF-κB activation is also increased in the stage of CP in CML compared with normal controls.^54^ By studying the p210-BCR-ABL-transformed interleukin 3-dependent DA1 cell line, it was found that BCR-ABL expression in this cell model abolished interleukin 3-dependent growth and that the inhibition of p65 by antisense oligonucleotides recovered the interleukin-3 dependence of BCR-ABL-transformed DA1 cells, suggesting that NF-κB may contribute to BCR-ABL-mediated tumorigenesis.^55^ A similar conclusion was drawn in the study of interleukin 3-dependent mouse myeloblast-like cell lines expressing BCR-ABL,^56^ and the activation of NF-κB is mediated by Ras. These data demonstrated the important role of NF-κB in the pathogenesis of Ph+ leukemia.

The NF-κB signaling pathway plays a key role in tumorigenesis. It promotes cell proliferation and survival, induces resistance to chemotherapy and mediates invasion and transformation.^57^ NF-κB activation was found to be involved in the occurrence of CML.^56,58,59^ Furthermore, NF-κB inhibitors have inhibitory effects on CML, and induces death of CML cells with the mutation of T315I Bcr-Abl,^54,60^ indicating that targeting NF-κB may be a valid avenue for the treatment of CML.

CBS and H_2_S have been confirmed to be involved in the development of various solid tumors, including colon, ovarian and liver cancer. Inhibition of CBS expression can inhibit the proliferation of tumor cells.^18,19,25^ In our study, we found that CBS and H_2_S are increased in bone marrow cells of CML patients. In addition, the expression of CBS was associated with the disease stages. We also confirmed the expression of CBS is increased in CML-derived K562 cells. As a chemical inhibitor of CBS, AOAA significantly reduced the proliferation of the primary mononuclear cells from the bone marrow of CML patients. These results provide new insight into the role of CBS in hematologic tumors. To further evaluate the translatable capacity of AOAA for its therapeutic effect, we injected C57BL/6 mice with different dosages of AOAA, which were converted from the concentrations used in the cell experiments. We found that treatment with 20 mg/kg AOAA, which was equivalent to the highest concentration (3.2 mM) in our cell study, did not cause any hematological, liver, and kidney toxicity in the mice (Supplementary Fig. S3).

In colon cancer, the inhibition of CBS can reduce the biological energy metabolism of tumor cells by reducing the production of endogenous H_2_S, thereby achieving an antitumor effect.^18^ By using sequence-specific shRNA to silence the expression of CBS or AOAA to inhibit its activity, we found that the inhibition of CBS not only reduced K562 cell proliferation, but also had pro-apoptotic and anti-migration effects in both cultured K562 cells and tumor xenografts. Besides CBS, AOAA has also been identified to inhibit some other enzymes including CSE and glutamic-oxaloacetic transaminase 1 (GOT1), which also play important roles in the regulation of cancer cell bioenergy.^28^ To further confirm the specific inhibitory effect of AOAA in our study, we applied AOAA to the CBS silenced cells and found AOAA significantly lost its anti-proliferative and pro-apoptotic effects in K562 cells. In addition, we also found that inhibition CBS reduced the level of H_2_S in K562 cells, followed by a decrease in the intracellular ATP production, which could be recovered by H_2_S donors. By examining the expression of apoptosis pathway-related proteins, we found that this effect is mediated through the activation of the mitochondrial apoptotic pathway. These results are consistent with the previous report that H_2_S stimulates mitochondrial energy metabolism mitogenesis.^18,33^

Inhibition of CBS and the reduction of H_2_S can increase ROS levels in ovarian cancer cells, inhibit the expression of the ROS-sensitive proteins p53 and p65 and reduce the activity of NF-κB to inhibit the proliferation of cancer cells.^19,61^ H_2_S can also affect the activity of NF-κB by affecting the thiolation level of cysteine^36^ of NF-κB^37^ or regulating GSH3 and IkB-α levels.^19,62^ Our results showed that the inhibition of CBS by AOAA or shRNA decreased NF-κB activity, resulting in transcriptional suppression of NF-κB-mediated downstream target gene expression. These results are consistent with the past study showing that H_2_S can affect NF-κB activity.^37^ Our study indicates that both CBS and NF-κB may be potential targets for anti-CML therapy.

Patients with Down syndrome have a significantly higher risk to suffer from myeloid leukemia, 10 to 20 times higher than those without Down syndrome, and they have lower possibility of developing solid tumors.^63,64^ The incidence of acute megakaryoblastic leukemia (AMKL) and acute lymphoblastic leukemia (DS-ALL) is 500 and 30 times higher in Down syndrome than in non-Down syndrome.^64–66^ The majority of Down syndrome patients are caused by presence of extra copy of chromosome 21, thus known as the trisomy-21 syndrome. CBS protein is encoded by the *CBS* gene located on the human chromosome 21. The expression of CBS in AMKL is approximately 200-times higher than that in the normal control group, DS cells also contain high H_2_S levels, which are associated with reduced mitochondrial complex IV activity and ATP generation.^67^ Thus, CBS/H_2_S may be involved in the pathogenesis of leukemia in trisomy 21 syndrome and targeting CBS/H_2_S may provide novel therapeutic approach for leukemia in DS patients.

Although our work has discovered the role of CBS in the CML, additional studies are required. Our clinical samples are from children, who have relatively low incidence of CML. However, previous studies have indicated that the age of patients is not related to the disease phenotype.^68^ To further confirm our findings, we need to expand the sample size and also explore the expression of CBS in adult CML patients. In addition, it was reported that AOAA did not induce cell cycle arrest but increased the G1 phase and reduced the G2 phase of HCT116 cells.^69^ However, our study found that shRNA-mediated CBS silencing induced S-phase arrest in K562 cells. The difference may be due to the heterogeneity of tumor cells. Thus, the role of CBS in various hematological tumors needs to be further explored in the future study.

## Materials and methods

### Materials

7-Azido-4-Methylcoumarin, L-cysteine, L-homocysteine, pyridoxal 5′-phosphate hydrate, 3-(4,5-dimethylthiazol-2-yl)-2,5-diphenyltetrazolium bromide (MTT), propidium iodide (PI), and ribonuclease (RNase) were purchased from Sigma-Aldrich (St. Louis, MO, USA). The following antibodies were purchased from Cell Signaling Technology (Beverly, MA, USA): rabbit anti-Bax, anti-PARP, anti-Cyto C, and mouse anti-caspase 9. Mouse anti-MPST was purchased from Santa Cruz. Rabbit anti-CBS and anti-CTH were obtained from HUA BIO and ZEN BIO, respectively. Mouse anti-β-actin was obtained from Sigma-Aldrich. A protease inhibitor cocktail was purchased from Roche (Basel, Kanton Basel, Switzerland). Lipofectamine 2000 transfection reagent was ordered from Invitrogen (Carlsbad, CA, USA). A FITC annexin V apoptosis detection kit was purchased from BD Pharmingen (San Diego, CA, USA). The Caspase-Glo® 3/7 Assay and the CellTiter-Glo® Luminescent Cell Viability Assay were purchased from Promega (Madison, WI, USA).

### Patient samples

Bone marrow samples from 17 children with newly diagnosed CML were collected at Children’s Hospital of Chongqing Medical University (Chongqing, China) from April 2014 to July 2016, and 24 age-matched and sex-matched children with nonmalignant hematologic diseases were collected in the same period as control group, and the bone marrow mononuclear cells and plasma were isolated. The diagnosis and staging of the patients were based on the “Guidelines for the Diagnosis of Chronic Myeloid Leukemia in China (2013 Edition)”. The patient characteristics are shown in Table 1. Four samples of the neonatal cord blood were collected in the Department of Obstetrics and Gynecology, the First Affiliated Hospital of Chongqing Medical University, and then CD34^+^ umbilical cord hematopoietic stem cells were extracted with a magnetic bead method following manufacture’s guideline (Miltenyi Biotec, Bergisch Gladbach, Germany).

### Cell culture and transwell cell migration assay

CML-derived K562 cells were grown in RPMI-1640 media (Gibco, Rockville, MD, USA) supplemented with 10% fetal bovine serum, penicillin (100 units/mL), and streptomycin (100 µg/mL) at 37 °C with 5% CO_2_ culture incubator. A 24-well transwell cell culture insert (Corning) with 8 μm wells was used for the transwell assay. Matrigel was added to the insert. After 4 h, shNT and shCBS-transfected cells starved for 16 h in serum-free medium were seeded in the upper chamber at a density of 1 × 10^6^/mL. After 24 h of incubation, non-migrating cells were removed from the top wells, while cells in the bottom wells were collected and counted in triplicate by trypan blue staining.

### RNA extraction and quantitative real-time PCR

Total RNA of bone marrow samples and cultured K562 cells was extracted by Blood (liquid sample) total RNA rapid extraction kit (centrifugal column type) (Bio Tek, Beijing, China) followed by the first-strand cDNA synthesis with PrimeScript™ RT reagent Kit (TaKaRa, Dalian, China). The PCR primers for CBS: forward 5’-gtaatcctgggaatggtgacg and reverse 5’-gaggcggatctgtttgaactg. The primers for CSE: forward 5’-gagcagttccatctcctattga and reverse 5’-ggcagcccaggataaataac. The primers for 3-MST: forward 5’-cgagacggcattgaacc and reverse 5’-ctggaacagatggcgga. The primers for β2-MG: forward 5’-ccagcgtactccaaagattca and reverse: 5’-gtcaacttcaatgtcggatgg. The relative mRNA expressions were detected with SYBR Premix Ex Taq II (TaKaRa, Dalian, China). The mRNA expressions were normalized to β_2_-MG, and 2-ΔΔCT method was used to analyze the relative gene expression.

### Protein extraction and immunoblot analysis

K562 cells treated with AOAA for 48 h and CBS-silenced cells were collected and lysed in RIPA buffer supplemented with a protease inhibitor cocktail (Roche Diagnostics). Nuclear and cytoplasmic protein was extracted using NE-PER^TM^ Nuclear and Cytoplamic Extraction (Thermo Scientific), Cytoplasmic proteins were used to detect Cyto C levels, while nuclear proteins were utilized for EMSA experiments. The cell lysates were separated with Tris-glycine-SDS-PAGE and transferred to polyvinylidene fluoride (PVDF-FL) membranes. Immunoblots were incubated with the corresponding antibodies.

### Cell viability and apoptosis assay

Cell viability was measured with an MTT assay. A total of 1 × 10^5^ cells were seeded in 96-well plates and treated with different concentrations of AOAA. After 12, 24, 36, and 48 h, 20 μL MTT (5 mg/mL) was added and incubated for 4 h at 37 °C. The cells were then incubated with 100 μL 20% SDS in dimethylamide/water (1:1, v/v; pH 4.7) until the formazan was fully dissolved. The absorbance of each well at 570 nm was measured on a Multiscan Spectrum. Apoptosis was detected according to the manufacturer’s instructions. Briefly, the cells were washed with cold phosphate-buffered saline (PBS) after each treatment and stained with 5 μL PI and 5 μL FITC-conjugated annexin V for 15 min at room temperature. The EGFP+sh-NT and sh-K562 cells were stained with APC-conjugated annexin. The fluorescence of cells was analyzed by flow cytometry (Becton Dickinson, NJ, USA). The annexin V-stained and PI-negative cells in the lower right quadrant of the plots represent the percentage of early apoptotic cells. The activities of caspase-3/7 in K562 CML cells were analyzed using Caspase-Glo® 3/7 Assay Systems according to the manufacturer’s protocol. The luminescence of each sample was detected in a Multiscan Spectrum.

### Tumor mouse model and drug treatment

3 × 10^7^ transfected K562 cells were subcutaneously injected into the armpits of BALB/c nude mice (6–8 weeks of age; SPF Biotechnology Co., Beijing, China). When the tumors were palpable, mice were randomly selected for intraperitoneal injection of PBS, AOAA (10 mg/kg.day) or AOAA (20 mg/kg.day) for 9 days. Tumor growth was measured every 3 days by using a dial caliper. Tumor volume were calculated with the equation: volume (V) = tumor mass length × width^2^/2.^70^ After mice were sacrificed, tumor xenografts were removed and measured. The xenografts were fixed in 10% formalin then embedded in paraffin. Sections (5-μm in thickness) were stained with hematoxylin and eosin (H&E) and visualized using light microscope.

### Methylene blue method for measuring the H_2_S concentration in bone marrow

One hundred microliter of bone marrow slurry was used from each sample. Then, 200 μL of zinc acetate (1%) was added, followed by 100 μL of N,N-dimethyl-1,4-phenylenediamine sulfate (20 mM, in 7.2 M HCl). After adding 133 μL of ferric chloride (30 mM, in 1.2 M HCl), the tubes were incubated at 37 °C for 30 min in the dark, then were centrifuged at 5000×*g* for 10 min, and 200 μL supernatant was placed in a 96-well plate. Finally, the plate was placed in a Multiscan Spectrum to detect the absorbance of each well at 670 nm.

### Measurement of cellular ATP levels

ATP concentration in cultured K562 cells was determined using CellTiter-Glo® Luminescent Cell Viability Assay kit (Promega, Madison, WI, USA). The luminescent signal reflecting ATP levels was recorded for 1 s by BioTek SynergyTM H1 microplate reader (BioTek., Winooski, VT, USA).

### Detection of H_2_S production in K562 cells

K562 cells were washed twice with PBS. Each cell pellet was lysed using 300 μL nondenaturating lysis buffer (50 mM Tris–HCl pH 8.0, 150 mM NaCl, 1% NP-40, and 1% Triton X-100) on ice for 1 h followed by centrifugation at 20,000×*g* for 5 min at 4 °C to sediment unlysed cells. The reaction mixture contained 300 μg of protein cell extract, 100 mM Tris HCl pH 8.0, 50 μM PLP, 10 mM L-cysteine, 0.5 mM L-homocysteine, and 10 μM of the fluorescent H_2_S probe 7-azido-4-methylcoumarin. After incubation for 2 h at 37 °C, the fluorescence of the H_2_S-specific probe was measured using a Synergy H1 Hybrid Microplate Reader (Bio Tek, VT, USA) using ex = 365 and em = 450 nm. Preliminary assays were carried out to ensure the linearity of fluorescence with respect to the amount of protein extracts. A standard curve for H_2_S was generated by adding NaHS to the fluorescent assay medium.

### Electrophoretic mobility shift assay (EMSA)

An NF-κB consensus oligonucleotide probe (5’-agttgaggggactttcccaggc) was labeled with IR700 dye (Bioneer Corporation, Daedeok-gu, Daejeon, Korea) and annealed with corresponding antisense oligos to generate a double-stranded probe at a final concentration of 0.01 pmol/μL. Binding reactions were performed according the manufacturer’s instructions (Odyssey® Infrared EMSA Kit) using 5 μg of total nuclear extraction with 0.01 pmol of oligonucleotides in a 10 μL reaction volume containing 1 μL 10× binding buffer, 1 μL 25 mM DTT, 0.5 μg poly[d(I-C)], at room temperature for 30 min. Binding reactions were resolved on a 4% Tris-glycine-EDTA gel. The gel was scanned using a LI-COR Odyssey (LI-COR Biosciences, Lincoln, NE, USA) at a wavelength of 700 nm. For cold-probe competition assays, a 1-fold, 5-fold, 15-fold, 20-fold, and 50-fold unlabeled probe was added as a competitor 30 min before the addition of the labeled probe. To confirm the specificity of the NF-κB-binding proteins, the reactions were supplemented with rabbit polyclonal antibodies against mouse p65 (CST) 30 min.

### Statistics

The number of times each experiment was repeated at least 3 times independently. For the data of normal distribution, the results are expressed by mean ± SEM and statistically analyzed by ANOVA or two-tailed *T* test. Multiple sets of non-normal data were compared using the Kruskall–Wallis *H* test, and subsequent pairwise comparisons were performed using the Nemenyi method. Values of *P* < 0.05 were considered significant.

### Study approval

Human subject study was approved by the institutional ethics board of Children’s Hospital of Chongqing Medical University. All subjects provided written informed consent for both study enrollment and sample collection. Animal experiments were conducted in accordance with Chongqing Medical University Animal Care and Use Committee.

## Supplementary information

Supplementary Figures

## Data Availability

The data that support the findings of this study are available from the corresponding author upon reasonable request.
