# Supplementary material for: Inhibition of cystathionine β-synthase promotes apoptosis and reduces cell proliferation in chronic myeloid leukemia
Source: Signal Transduct Target Ther. 2021 Feb 8;6:52. doi: 10.1038/s41392-020-00410-5 (PMC7870845; doi:10.1038/s41392-020-00410-5)
Supplement: Supplementary file 1 — Supplementary Figures [file 41392_2020_410_MOESM1_ESM.docx]

Supplementary Materials for

**Inhibition of cystathionine β-synthase promotes apoptosis and reduces cell proliferation in chronic myeloid leukemia**

Dan Wang ^1,#^, Huan Yang ^1,#^, Yun Zhang ^2,#^, Rong Hu ^1^, Dongjie Hu ^1^, Qunxian Wang ^1^, Yannan Liu^1^, Mingjing Liu^1^, Zijun Meng^1^, Weihui Zhou^1^, and Weihong Song^1,2,*^

^1^ Chongqing City Key Lab of Translational Medical Research in Cognitive Development and Learning and Memory Disorders; Ministry of Education Key Laboratory of Child Development and Disorders; National Clinical Research Center for Child Health and Disorders (Chongqing); and Department of Neurology, Children’s Hospital of Chongqing Medical University, Chongqing 400014, China

^2^ Townsend Family Laboratories, Department of Psychiatry, The University of British Columbia, 2255 Wesbrook Mall, Vancouver, BC V6T 1Z3, Canada

**^#^** These authors contributed equally to this work.

Correspondence to: [weihong@mail.ubc.ca](mailto:weihong@mail.ubc.ca)

**This PDF file includes:**

Figures. S1 to S3

**
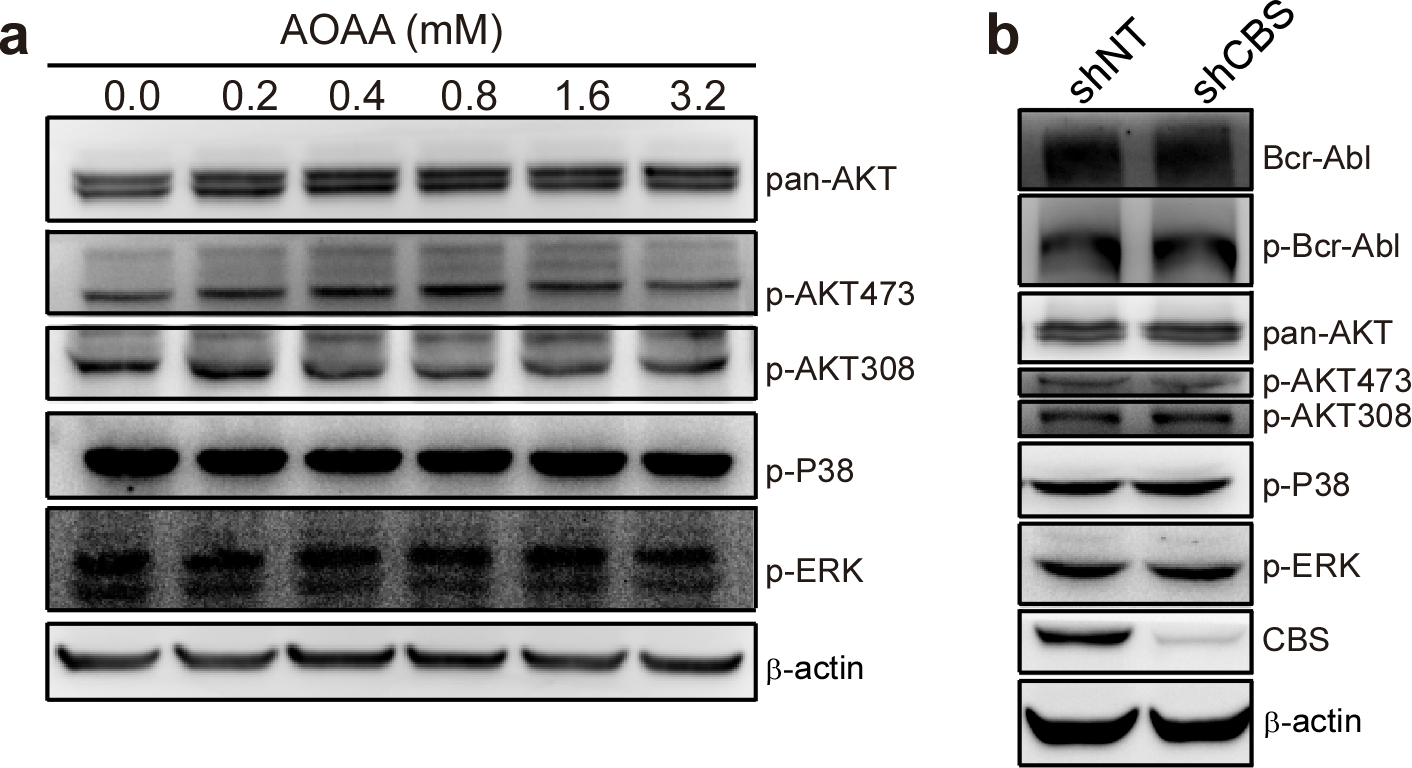
**

**Supplementary Figure S1.** **The effect of CBS inhibition on BCR-ABL signaling pathways.** **(A)** K562 cells were treated with AOAA (0.0, 0.2, 0.4, 0.8, 1.6 and 3.2 mM) for 48h. Western blot analysis was performed to detect pan-AKT, phosphorylated AKT, P38 and ERK expressions. **(B)** Protein levels of CBS, BCR-ABL, phosphorylated BCR-ABL, AKT, P38 and ERK were detected by Western blot analysis in shNT- and shCBS-K562 cells.


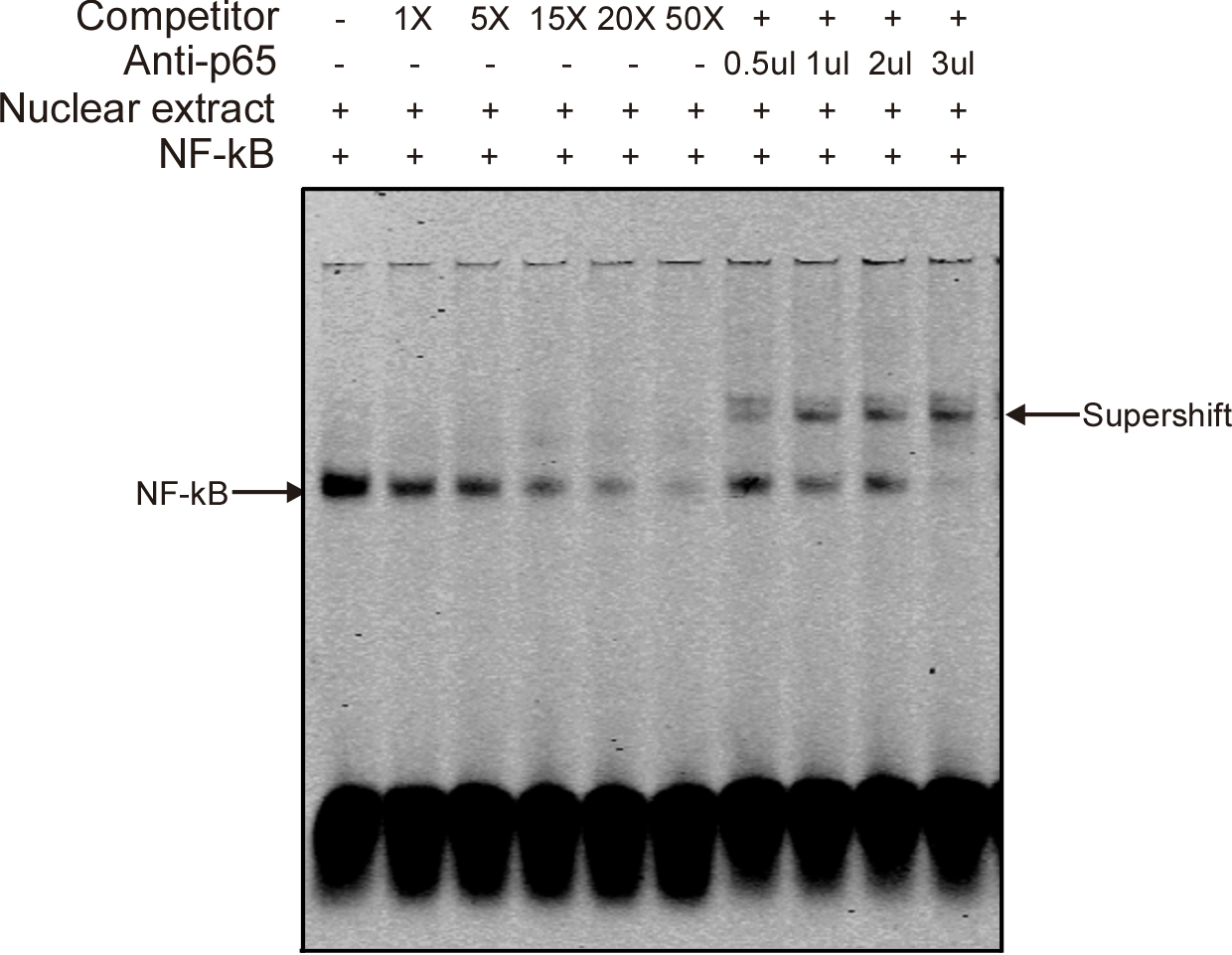


**Supplementary Figure S2.** **Cold-probe competition and supershift EMSA assay.** In cold-probe competition assays, 1-, 5-, 15-, 20- and 50-fold unlabeled probes were used as competitors and applied 30 min before adding the labeled probe. For the supershift test, 0.5 μl of rabbit anti-p65 antibody (1 μl, 2 μl or 5 μl) was added 30 min before mixing with nuclear protein.


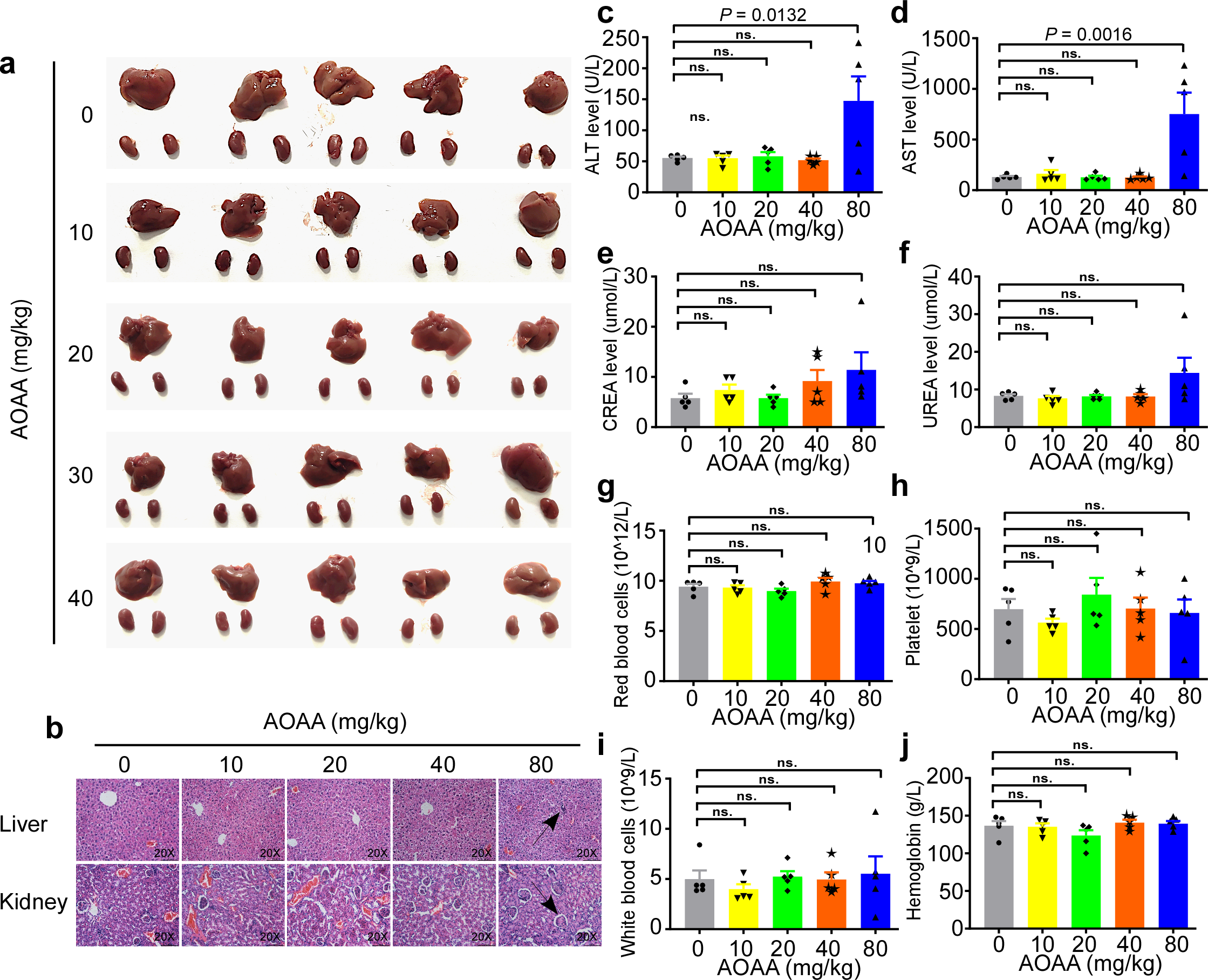


**Supplementary Figure S3.** Evaluation of the toxic effect of AOAA treatment in mice. Mice were subjected to intraperitoneal injection of AOAA (10,20, 40 and 80 mg/kg). The same volume of PBS was used as a solvent control. On the third day, mice were euthanized to collect blood and tissues for measurement, (**A**) Morphology of liver and kidney tissues. (**B**) Liver and kidney histological pictures from mice treated with AOAA or vehicle. The black arrow in the upper row points to perilobular inflammation, the black arrow in the lower row points to the mesangial enlargement. **(C-F)** Effect of AOAA treatment on plasma markers of organ damage in mice. **(G-J)** blood routine examination of mice treated with different concentrations of AOAA. Data are shown as mean ± SEM of n = 5 mice per group.
